# Supplementary material for: Cholesterol and Sphingomyelin Polarize at the Leading Edge of Migrating Myoblasts and Involve Their Clustering in Submicrometric Domains
Source: Biomolecules. 2023 Feb 7;13(2):319. doi: 10.3390/biom13020319 (PMC9953279; doi:10.3390/biom13020319)
Supplement: Supplementary file 1 [file biomolecules-13-00319-s001.zip › biomolecules-2176342-supplementary.pdf]

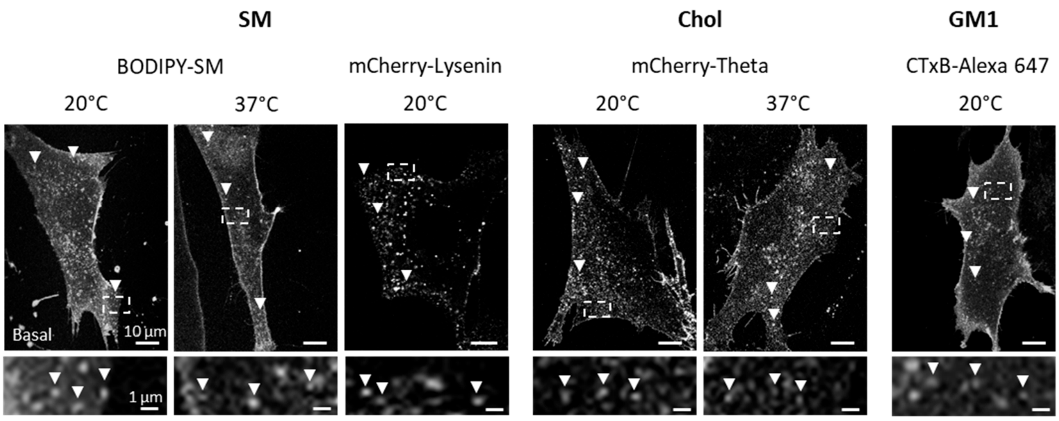

Figure S1. Clusters enriched in sphingomyelin, cholesterol or GM1 ganglioside are revealed at the outer plasma membrane of C2C12 myoblasts, no matter the temperature of labeling. Basal confocal sections of cells single-labeled for SM (BODIPY-SM or mCherry-lysenin), chol (mCherry-theta) or GM1 (CTxB-Alexa 647) at 20 °C or 37 °C. Dotted rectangle, close-up area. White arrowheads, lipid clusters. Representative of 1 experiment.

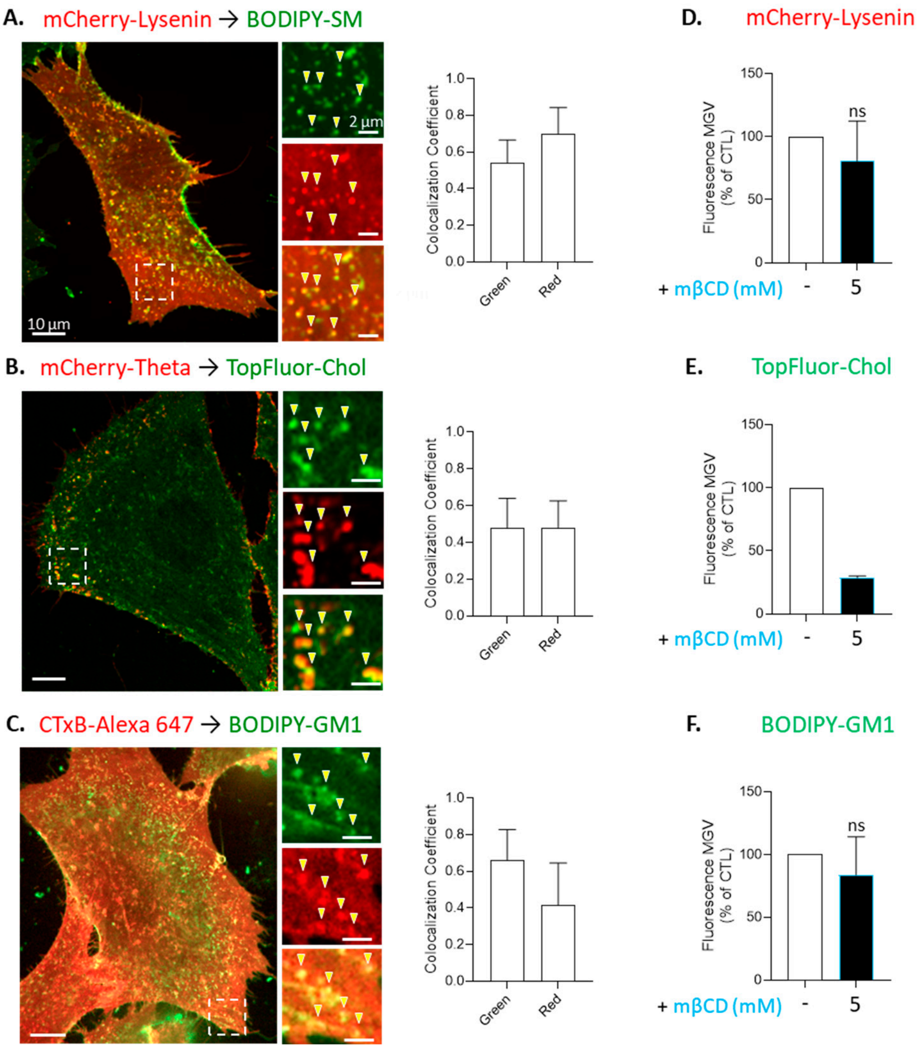

Figure S2. Lipid analogs and toxin fragments represent relevant and complementary probes to evidence lipid clusters. (**A**–**C**) Using two different probes, cells were double-labeled in a sequential manner for SM (lysenin then BODIPY-SM, (**A**), chol (theta then TopFluor-Chol, (**B**) or GM1 (CTxB then BODIPY-GM1, (**C**). Colocalization in domains was highlighted in insets (arrowheads) and the extent of colocalization on the entire cell surface was quantified using green or red colocalization coefficients (colocalization of the lipid analogs with the toxins or inversely) (means ± SD, n = 8–14

cells from 1–2 independent experiments). (**D**–**F**) After pretreatment or not with mβCD, cells were single-labeled for SM (lysenin), chol (TopFluor-Chol) or GM1 (BODIPY-GM1) then analyzed for their fluorescence intensity using MGV. Data are expressed as % of control (means ± SD, n = 2–4 independent experiments). One sample Wilcoxon test. Statistics above the columns refer to the corresponding control.

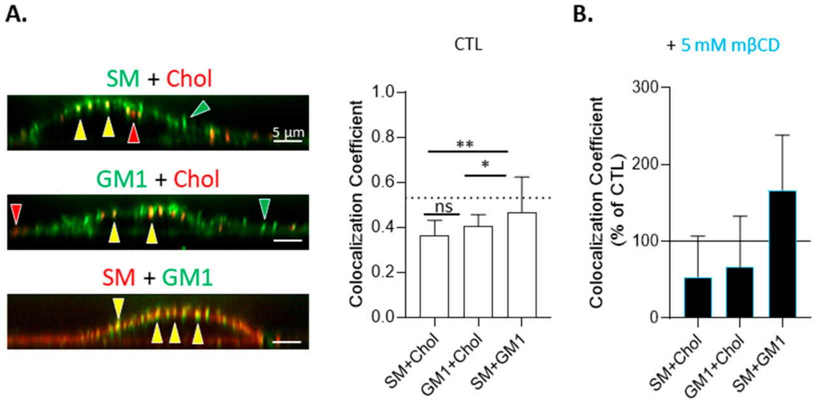

Figure S3. The extent of colocalization between sphingomyelin and GM1 ganglioside is higher than between sphingomyelin or GM1 ganglioside with cholesterol and is specifically increased by methyl-β-cyclodextrin. Resting cells were either directly double-labeled for SM/chol, GM1/chol or SM/GM1 (BODIPY-SM or lysenin, theta, BODIPY-GM1) (**A**) or pretreated with mβCD then double-labeled (**B**). Colocalization was then quantified using the green colocalization coefficient. Red and green arrowheads, domains enriched in one type of lipid; yellow arrowheads, colocalization; dotted line, reference colocalization value from the labeling of SM using two SM different probes (from Figure S2A); straight line, control. Data are expressed as the sum of the colocalized pixel count divided by the sum of the colocalized and the green pixel counts (means ± SD; n = 4–15 cells from 2–8 independent experiments). Kruskal-Wallis test. Statistics between different groups are indicated with bars on top of the graphs.

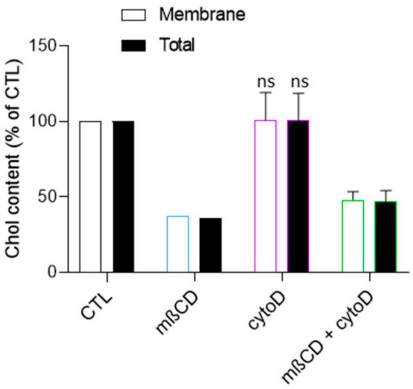

Figure S4. Membrane and total cholesterol contents are not affected by cytochalasin D treatment. Cells were treated with 5 mM mβCD, 0.5 µM cytoD or a combination of both treatments, then assessed for membrane chol and total chol content by addition of chol esterase. Data expressed as means ± SD (n = 1–3 independent experiments). Kruskal-Wallis test. Statistics above the columns refer to the corresponding control.

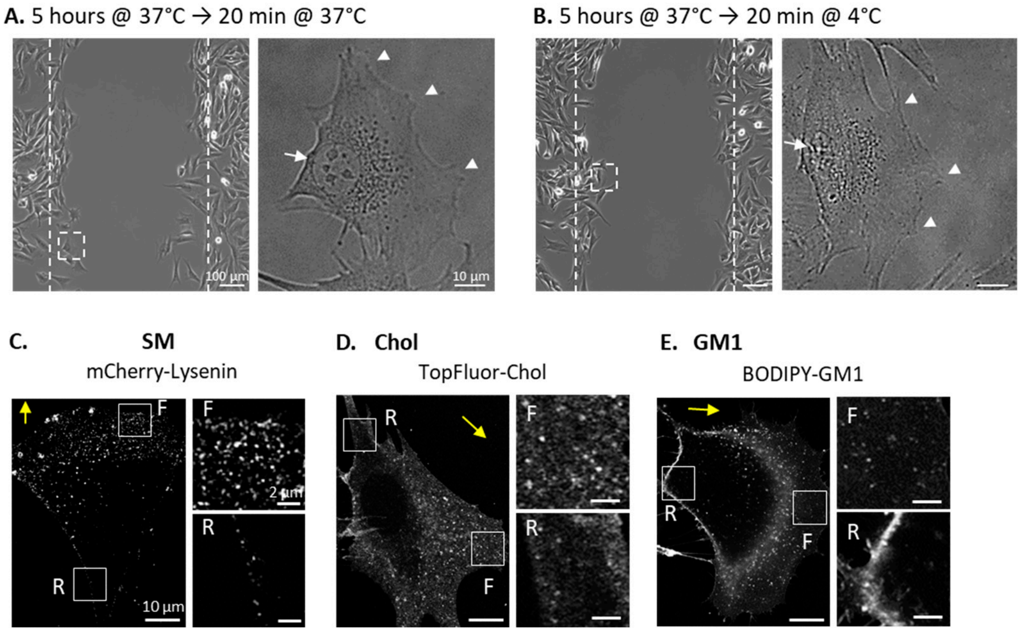

Figure S5. Temperature of labeling does affect neither myoblast migration nor morphology and sphingomyelin and cholesterol polarize at the leading edge in contrast to the GM1 ganglioside. (**A**,**B**) Cells were visualized by phase contrast microscopy after 5 h of migration at 37 °C followed by 20 min incubation at 37 °C (**A**) or at 4 °C (**B**). Dotted lines, starting point of migration; arrows, nucleus localization at the rear; arrowheads, protrusions of the migration front. Representative of 1 independent experiment. (**C**–**E**) After 5h of migration in Ibidi chambers, cells were single-labeled for SM (lysenin), chol (TopFluor-Chol) or GM1 (BODIPY-GM1) to visualize lipid polarization. Yellow arrows, direction of migration; F, migration front; R, rear. Representative of > 5 independent experiments for each probe except TopFluor-Chol (n = 1).

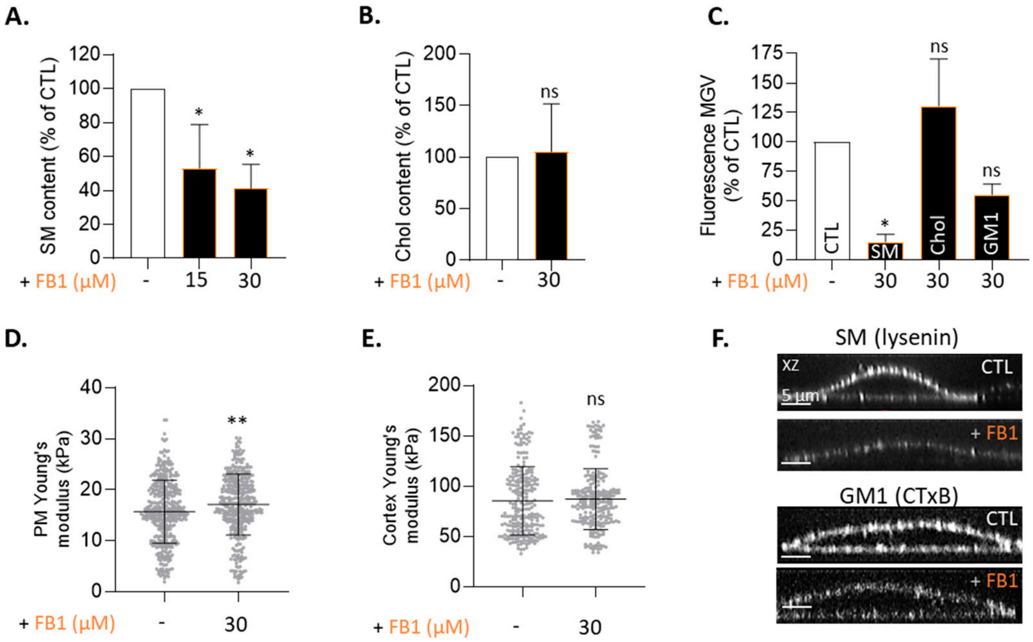

Figure S6. Sphingolipid synthesis inhibition by fumonisin B1 specifically decreases sphingomyelin content, sphingomyelin and GM1 surface labeling and domains. (**A**–**C**) Myoblasts were treated with the indicated concentrations of FB1. Cells were then assessed for SM (n = 4–5 independent experiments; **A**) or chol content (n = 3 independent experiments; **B**), or single-labeled for SM, chol or GM1 (lysenin, theta, CTxB) to quantify basal fluorescence MGV (n = 4 independent experiments; **C**). Data are expressed as % of control (means ± SD). Kruskal-Wallis test followed by Dunn's multiple comparisons test. (**D**,**E**) Plasma membrane and cortex rigidity of myoblasts left untreated or treated with FB1 evaluated by AFM. Data are expressed as means ± SD (n = 6 cells from 1 experiment with 50

measures/cell). Unpaired *t*-test. (**F**) Cells were treated with 30 µM FB1 then labeled for SM (lysenin) or GM1 (CTxB) and visualized by confocal microscopy with XZ reconstructions. Statistics above the columns refer to the corresponding control.

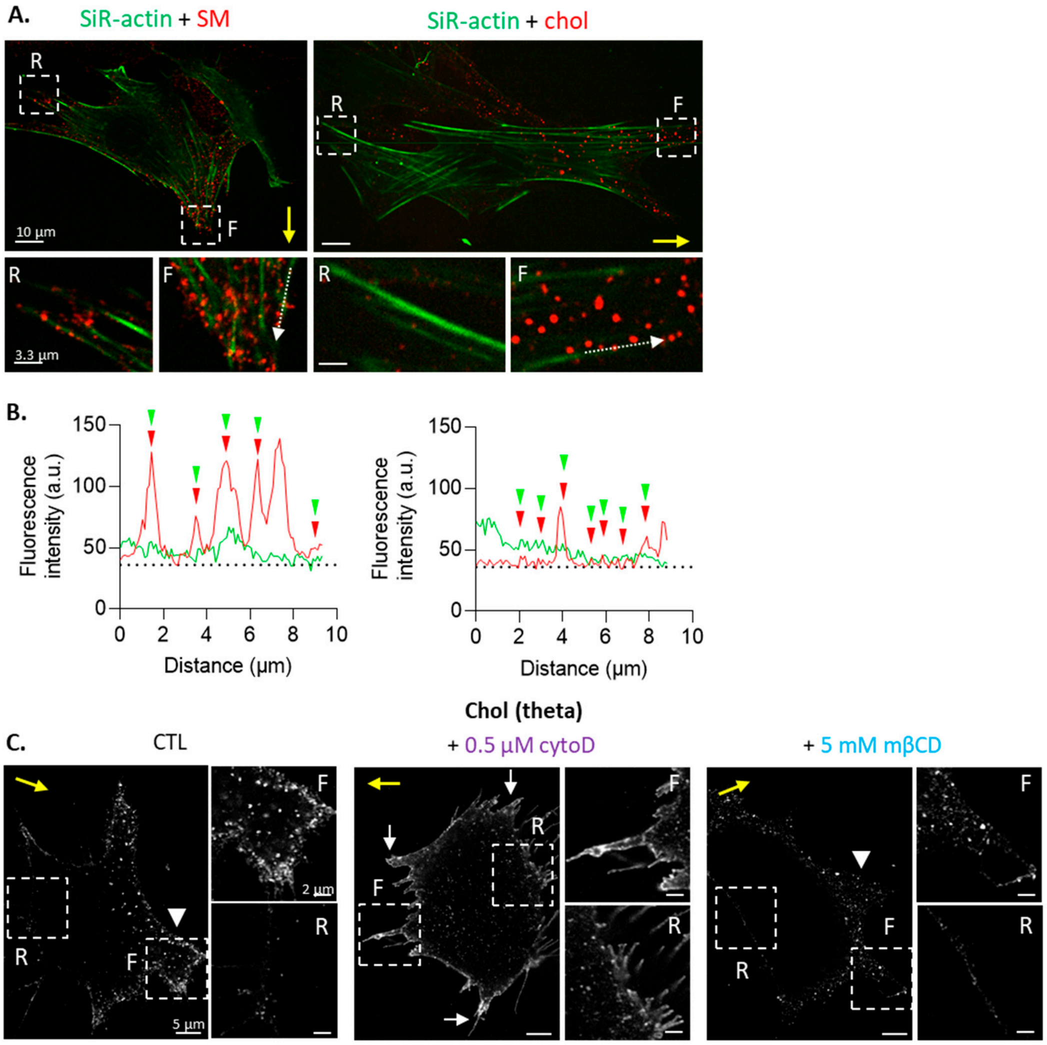

Figure S7. Sphingomyelin- and cholesterol-enriched domains show proximity with F-actin stress fibers at the migration front and the clustering of chol-enriched domains in lamellipodia is differentially impaired upon cholesterol depletion and cytoskeleton impairment. (**A**,**B**) Cells migrated for 5 h in Ibidi chambers then were double-labeled for actin (SiR-actin) and SM (lysenin, n = 2 independent experiments) or chol (theta, n = 3 independent experiments) (**A**). Proximity analysis was performed using fluorescence intensity profiles (**B**). Yellow arrows, direction of migration. R, rear; F, front. Dotted arrows, cell sections for profile analysis in B. Dotted line, treshold. Green and red arrowheads, close proximity between lipid and F-actin. (**C**) Cells were pretreated or not with cytoD or mβCD, migrated for 5 h in Ibidi chambers and then single-labeled for chol (theta). Yellow arrow, direction of migration; F, front; R, rear; white arrowheads, lamellipodia; white arrows, filopodia. Representative images of 4 independent experiments.

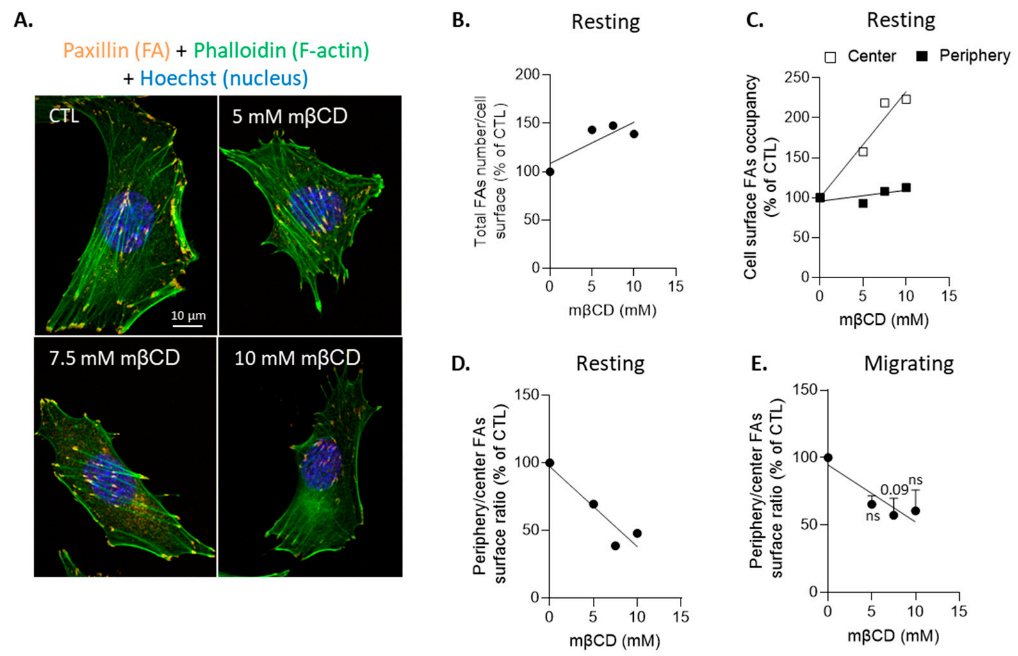

Figure S8. Focal adhesion distribution at the myoblast periphery vs. the center shows a tendency to decrease upon cholesterol depletion, as revealed both on resting and migrating myoblasts. Cells were pretreated with the indicated concentrations of mβCD then (immuno)labeled for F-actin (green), paxillin (orange) and nuclei (blue). (**A**–**D**) Resting myoblasts. (**A**) Representative images on resting cells. (**B**–**D**) Quantification of FA total number relative to cell surface (**B**), cell surface occupancy in the center and periphery of the cell (**C**), and corresponding periphery/center FA surface occupancy ratio (**D**). Data expressed in % of control (n = 1 experiment). (**E**) Migrating myoblasts. Cells were pretreated as in (**A**–**D**) then migrated for 5 h and periphery/center FA surface occupancy ratio was quantified. Data expressed in % of control (n = 3 independent experiments). Kruskal-Wallis followed by Dunn's multiple comparisons test. Statistics above error bars refer to the corresponding control.

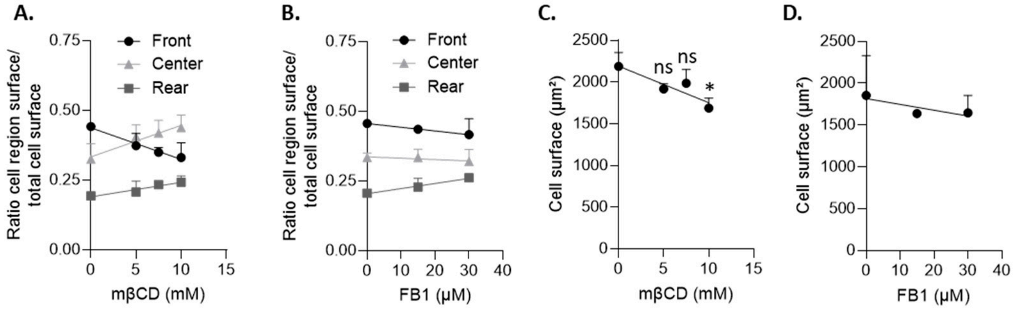

Figure S9. The cell migration front is decreased upon methyl-β-cyclodextrin treatment in contrast to sphingolipid treatment. Cells were pretreated with increasing concentrations of mβCD (**A**,**C**) or FB1 (**B**,**D**) then migrated for 5 h in Ibidi chambers. Cells were then (immuno)labeled for F-actin, paxillin and nuclei (data from Figure 8). Cell surfaces at the front, center and rear of the cell were extracted during analysis and plotted in A,B. Data are expressed as the ratio of the cell region surface vs. the total cell surface, which was also extracted during analysis and plotted in C,D (means ± SD, n = 2–3 independent experiments). Kruskal-Wallis test followed by Dunn's multiple comparisons test. Statistics above error bars refer to the corresponding control.

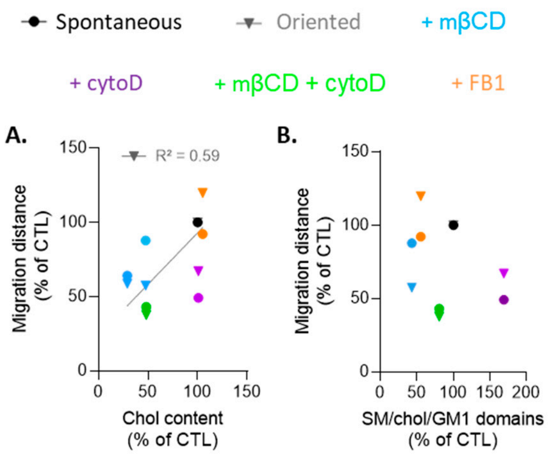

Figure S10. The cholesterol content, but not SM/chol/GM1-enriched domain abundance, slightly correlates with oriented myoblast migration. Relations between chol content (**A**) and SM/chol/GM1-enriched domains (**B**) with myoblast spontaneous (circles) and oriented (triangles) migration. Regression and R^2 are indicated only if > 0.5.
